# Interpretation of illness in cancer survivors is associated with health-related variables and adaptive coping styles

**DOI:** 10.1186/1472-6874-9-2

**Published:** 2009-01-29

**Authors:** Arndt Büssing, Julia Fischer

**Affiliations:** 1 Chair of Medical Theory and Complementary Medicine, Faculty of Medicine, University of Witten/Herdecke, Gerhard-Kienle-Weg 4, 58313 Herdecke, Germany

## Abstract

**Background:**

A patient's interpretation of illness may have an influence on the choice of coping strategies and decision making. We intended to analyze the meaning German cancer survivors would attribute to their disease, and investigated intercorrelations between the respective interpretations, health-related variables and adaptive coping strategies.

**Methods:**

In an anonymous cross-sectional survey, we analyzed the interpretations of disease (according to Lipowski's eight 'meaning of illness' categories) in 387 patients with cancer (81% breast cancer). To make statements about their conceptual relationships with health-related variables, we correlated the 8 items of the 'Interpretation of Illness' questionnaire (IIQ) with health-related quality of life, anxiety/depression, fatigue, life satisfaction, and adaptive coping strategies.

**Results:**

Most cancer survivors regarded their disease as a challenge (52%), others as value (38%) or even an interruption of life (irreparable loss; 35%); weakness/failure (5%) and punishment (3%) were rated the lowest. The fatalistic negative interpretations 'interruption/loss' and 'enemy/threat' were inversely correlated with mental health-related quality of life and life satisfaction, and positively with an escape-avoidance strategy, depression and anxiety. In contrast, positive disease interpretations (i.e., 'challenge' and 'value') correlated only with adaptive coping strategies. Physical health correlated with none of the disease interpretations.

**Conclusion:**

Despite conceptual limitations, the 8-item schema could be regarded as a useful screening approach to identify patients at risk for reduced psychosocial functioning.

## Background

Among the numerous ways to cope with disease, two general strategies can be distinguished: problem-solving (i.e., do something active to avoid stressful circumstances) and emotion-focused coping strategies (i.e., try to regulate the emotional consequences of stressful or potentially stressful events). Folkman and Lazarus found that both types were used to face stressful situations [1]. Carver et al. differentiated active and avoidant coping strategies, among them 'Resignation/Acceptance' (i.e., accepting the fact that the stressful event has occurred and is real) and 'Focus on and Venting of Emotions' (i.e., increased awareness of one's emotional distress, and concomitant tendency to ventilate or discharge those feelings) [2]. An active coping means to change the nature of the stressor itself or how one thinks about it. In contrast, avoidant strategies are intended to prevent a direct confrontation with stressful events, and may often result in inappropriate activities such as alcohol abuse or depressive states. These avoidance strategies were identified as psychological risk factors or marker for adverse responses to stressful life events [3].

In cancer patients, Derks et al. found that younger individuals used active coping strategies significantly more often and they perceived more internal control over the cause of their disease; in contrast, older patients used religious coping and religious control more frequently at all assessments; in both groups, avoidance coping was associated with more depressive symptoms and a worse quality of life [4].

However, the emotional responses to illness and modes of coping with illness are influenced by the personal perception (interpretation) of illness [5]. The subjective meaning of illness is influenced by intrapersonal, disease-related and environmental factors. These interpretations of illness may have an influence on preferences in decision-making and choice of coping strategies, which may change within time and course of disease. From a clinical point of view, changing negative illness interpretations and depressive or avoidance coping by means of an intervention and encouraging social support by means of patient support groups may at least improve quality of life.

Based on early descriptive work by the psychiatrist Zbigniew J. Lipowski (1924–1997), eight categories of meaning which would influence the choice of coping strategies were devised as prevalent in our culture, i.e., challenge, value, enemy, punishment, weakness, loss, relief, and strategy [6]. Degner et al. initiated a research model on decision-making [7] which implicated the 'meaning of illness' as developed by Lipowski. In their study enrolling 1,012 Canadian women with breast cancer at various points after diagnosis [7], the majority chose 'challenge' (57%) or 'value' (28%) to describe the meaning of breast cancer, whereas only a few chose 'enemy' (8%) or 'irreparable loss' (4%). At follow-up assessment 3 years later, women who ascribed a negative meaning of illness with choices such as 'enemy', 'loss,' or 'punishment' had significantly higher levels of depression and anxiety and poorer quality of life than women who indicated a more positive meaning [7]. Also in patients from Great Britain, 'challenge' was chosen most often (62%), followed by 'value' (14%) and 'enemy' (13%) [8]. In a population of 187 Swedish breast cancer patients, 'challenge' was chosen most often (33%); the few patients with disseminated cancer chose 'enemy', 'punishment', 'weakness' and 'irreparable loss' more often than patients in the earlier stages of disease [9]. In a cross-sectional study enrolling 313 patients with chronic pain conditions from Germany, both 'challenge' and the negative rating 'adverse interruption' (loss) were rated the highest, while 'punishment' was rated the lowest [10]. Also in 405 patients with renal diseases (predialysis, haemodialysis and transplant) recruited in the North of England, 'challenge' was selected by most patients (63%), with similar results in all three patients groups; those selecting 'challenge' and 'value' appeared to have a more positive outlook than other patients[11]. The authors concluded that the 8-item schema appeared to be comprehensive, but is in need of further refinement [11].

The aim of this analysis was first to investigate the meaning German cancer survivors attribute to their disease (in terms of a positive or negative interpretation), and second to analyze intercorrelations between the respective interpretations, health-related variables and adaptive coping strategies. Our hypothesis was that particularly the positive interpretations are related to better health, higher quality of life and life satisfaction, while the negative interpretations are related to more depressive states and coping strategies.

## Methods

### Patients

In an anonymous cross-sectional survey, we analyzed the data of German cancer survivors, recruited at a conference of breast cancer support groups in August 2007 in Magdeburg (East-Germany). All subjects were informed of the purpose of the investigation, were assured of confidentiality, gave informed consent to participate, and completed the anonymous questionnaire (which neither asked for names, initials, addresses or clinical details – with the exception of a diagnosis) by themselves. Four-hundred questionnaires were handed out to patients consenting to participate, and within 3 days we received 396 questionnaires back.

### Demographic characteristics

Among 396 participants, 387 responded to the 'Interpretation of Illness Questionnaire' (IIQ), and thus were enrolled in this analysis. Their mean age was 59.7 ± 7.3 years; however, one individual was male. As shown in Table 1, the majority had breast cancer (81.4%), 3.6% colorectal cancer, 3.4% ovary cancer, and 11.6% other. The mean duration of disease was 10.9 ± 6.4 years. Eighty patients had a relapse, 41 patients mentioned metastases. Most patients were married and had a low or intermediate educational level (Table 1). A Christian affiliation was predominating (67.8%), 30.9% had no religious denominations, and 5 patients (1.3%) did not reply to this question.

**Table 1:** Demographic and psychometric data of 387 cancer patients

	**Scores of all patients**	***Interpretation***
**mean age **(years)	59.7 ± 7.3	

**family status **(%)		
married	69.1	
living with partner not married with living	2.6	
alone	9.5	
divorced	7.7	
widowed	11.1	

**educational level **(%)		
secondary (Hauptschule)	35.6	
junior high school (Realschule)	34.8	
high school (Gymnasium)	17.8	
other	10.7	

**mean duration of disease **(months)	10.9 ± 6.4	

**cancer localization **(%)		
breast	81.4	
colorectal	3.6	
ovarian	3.4	
other	11.6	

**body mass index categories **(%)		
< 19	0.5	underweight
19–24	21.7	normal weight
25–30	55.6	overweight
> 30	22.2	obesity

**SF-12's health-related quality of life**		
physical health	42.7 ± 9.7	reduced
mental health	47.0 ± 10.1	slightly reduced

**HADS**		
anxiety	6.7 ± 3.4	not relevant
depression	4.5 ± 3.4	not relevant

**fatigue sum score**	25.4 ± 9.6	possible fatigue

**life-satisfaction sum score**	75.7 ± 15.9	high

**adaptive coping strategies**		
conscious and healthy living	81.3 ± 12.1	very high
positive attitudes	77.5 ± 15.6	high
reappraisal: illness as chance	61.9 ± 24.8	moderately/high
trust in medical help	79.6 ± 22.2	high
search for information/alternative help	72.2 ± 18.7	high
trust in god's help	56.6 ± 34.8	moderately

Results are mean values or relative proportion, respectively

### Measures

The instrument to measure the 'Meaning of Illness' was a German language 8-item questionnaire based on the work of Lipowski, the 'Interpretation of Illness Questionnaire' (IIQ). The following eight categories were used: "I regard my illness as .... a challenge, something of value, a threat (enemy), a punishment, a weakness of my own, an adverse interruption of my life (loss), a relieving break from the demands of life (relief), a call for help (strategy)".

The items were scored on a 5-point Lickert scale from disagreement to agreement (0 – does not apply at all; 1 – does not truly apply; 2 – don't know; 3 – applies quite a bit; 4 – applies very much), and are referred to a 100% level (4 – applies very much = 100%).

Because the items were never approved by reliability and factor analysis in patients with cancer (except by a grouping of meaning categories [7,9]), we performed an exploratory analysis first. The reliability of the IIQ was evaluated with internal consistency coefficients, which reflect the degree to which all items on a particular scale measure a single (uni-dimensional) concept. To combine several items with similar content, we relied on the technique of factor analysis, which examines the correlations among a set of variables, in order to achieve a set of more general factors. VARIMAX-factor analysis was repeated rotating different numbers of items in order to arrive at a convergent solution embodying both the simplest structure and the most coherent.

### Health related measures

To test the external validity of the single items and the IIQ, and to make statements about the conceptual relationships with health-related variables, we enrolled several other instruments:

Physical and mental health-related quality of life was measured with the *Medical Outcomes Study Short-Form Health Survey SF-12 *which differentiates physical and mental health [12,13]. Life satisfaction was measured with a modification of Huebner's *Brief Multidimensional Students' Life Satisfaction Scale *[14], which addresses the following aspects: family life, friendships, work, myself, where I live, overall life, and two additional items, i.e., financial situation and future perspectives. This *Brief Multidimensional Life Satisfaction Scale *(BMLSS) was recently validated in elder patients (Büssing et al [14]., submitted for preparation) and thus can be used in this context.

Because fatigue may often occur as depression [15], we measured anxiety and depression with the *Hospital Anxiety and Depression Scale *(HADS) [16,17], and differentiated physical, cognitive and affective fatigue with the *Cancer Fatigue Scale *(CFS-D) [18]. For this CFS-D, a maximal sum score of 60 could be achieved (score ≥ 30 represent fatigue, scores between 23 and 29 represent possible fatigue, while scores ≤ 22 are without any relevance).

Active and adaptive coping styles, such as to create favorable conditions, search for information, medical support, religious support, social support, initiative spirit, and positive (re)interpretation of disease were measured with the *AKU questionnaire *[10,19]. This instrument refers to the concept of an internal respectively an external locus of disease control, and differentiates Conscious Way of Living (intrinsic), Positive Attitudes (intrinsic), Trust in Medial Help (external), Trust in God's Help (external), Search for Information and Alternative Help (external), and Reappraisal: Illness as Chance (intrinsic; appraisal style). The scores were referred to a 100% level (scores > 50% represent a positive attitude, while scores < 50% represent a negative attitude).

The AKU bears an independent 3-item scale termed *Escape from illness *which is an indicator of an avoidance-escape strategy to deal with illness (i.e., "fear what illness will bring", "would like to run away from illness", "when I wake up, I don't know how to face the day"). It was confirmed recently that this scale correlated strongly with depression, and negatively with life satisfaction [20]. The items were scored on a 5-point Lickert scale from disagreement to agreement, and referred to a 100% level.

### Statistics

Data were presented as mean values ± standard deviations or relative proportions (%). Reliability and factor analysis, analyses of variance (ANOVA) and correlation analyses were performed with SPSS 15.0 for Windows (SPSS GmbH Software, Munich). We judged p < 0.05 as significant.

## Results

### Principal component analysis of the interpretations of disease

As shown in table 2, the IIQ had a satisfactory internal consistency (Cronbach's alpha = 0.730). The item difficulty index (1.51 [mean value]/4) was 0.38; just one item ('weakness') was below the acceptable range of 0.2 to 0.8, indicating a bottom effect. Primary factor analysis pointed to a 3-factor solution (which explains 69% of variance) with one 4-item factor (eigenvalue 2.9) and two 2-item factors (eigenvalues 1.5 and 1.1, respectively (Table 2). Among four negative items, the items 'interruption of life/loss' and 'enemy/threat' on the one hand and the items 'weakness/failure' and 'punishment' on the other hand, loaded on different factors, indicating a differential interpretation of these perceptions by the patients. Although 'relieving break' and 'call for help' would load with 'weakness/failure' and 'punishment' on a single factor, they nevertheless share relevant side-loadings with the positive items. One may conceptualize the former motifs both as a strategy.

**Table 2:** Mean values, reliability parameters and factor loading

	**Means ± SD **[0–4]	**Difficulty index **(= 1.51)	**Corrected Item-Total Correlation**	**Alpha if Item Deleted **(alpha = 0.730)	Factor loadings *		
Weakness/failure	0.77 ± 1.01	0.19	.474	.695	**.876**		
Punishment	0.78 ± 0.96	0.20	.510	.691	**.823**		.264

Relieving break	1.27 ± 1.22	0.32	.559	.674	**.594**	.460	
Call for help/strategy	1.60 ± 1.30	0.40	.455	.695	**.529**	.373	

Value	1.96 ± 1.35	0.49	.327	.724		**.848**	
Challenge	2.34 ± 1.32	0.59	.357	.716		**.830**	

Loss/interruption	1.88 ± 1.29	0.47	.318	.724			**.897**
Enemy/threat	1.45 ± 1.23	0.36	.443	.698	.272		**.818**

* Principal Component Analysis; Varimax Rotation with Kaiser Normalization (rotation converged in 5 Iterations).

However, a core criteria for extracting factors, i.e., at least 3 items with significant loadings (> 0.30), is not fulfilled for 2 of the 3 factors. Even a reduction of the number of extracted factors resulting in a 2-factor solution (which explained 55.5% of variance) with 4 'negative' items and 4 'positive' items was less convincing. Here, 'relief' and 'strategy' would load now on the positive scale, but with strong side-loadings on the negative scale. We therefore decided to perform the further analyses with the respective single items rather than the putative IIQ scales.

### Interpretation of illness by cancer patients

Among the cancer survivors, 52% regarded their disease as a 'challenge', 38% as 'value', 35% as an 'interruption of life/irreparable loss', 28% as a 'relieving break', 27% as a 'call for help' 21% as an 'enemy/threat', 5% as own 'weakness/failure', and 3% as a 'punishment'.

As shown in table 3, age had a significant impact on the interpretation 'punishment' (stronger rejection in younger patients than in elderly), while neither the family status nor the educational level, duration of disease or the Body Mass Index (which may be negatively associated with health-related quality of life [physical: r = -0.31, p < 0.001], and thus could have an influence on disease interpretation) had a significant impact (data not shown). 'Punishment' was rejected stronger in patients without a Christian denomination, while patients with a Christian denomination had positive ratings for 'challenge', which was low in patients without any denominations (Table 3).

**Table 3:** Mean values with respect to demographic data (analyses of variance)

		**Weakness/failure**	**Punishment**	**Relieving break**	**Call for help/strategy**	**Value**	**Challenge**	**Interrupt/loss**	**Enemy/threat**
		*guilt-associated negative*		*strategy-associated*		*positive*		*fatalistic negative*	

All patients	Mean	0.77	0.78	1.27	1.60	1.96	2.34	1.88	1.45

	SD	1.01	0.96	1.22	1.31	1.35	1.32	1.29	1.23
< 50 years	Mean	0.73	0.51	1.09	1.47	1.93	2.60	2.09	1.62
	SD	1.07	0.94	1.18	1.36	1.48	1.34	1.33	1.44
50–60 years	Mean	0.70	0.78	1.33	1.76	2.14	2.51	1.80	1.44
	SD	0.98	0.95	1.28	1.35	1.37	1.28	1.25	1.22
61–70 years	Mean	0.81	0.84	1.27	1.52	1.81	2.13	1.90	1.42
	SD	1.01	0.98	1.20	1.28	1.31	1.34	1.34	1.20
> 70 years	Mean	0.92	0.86	1.31	1.38	2.00	2.29	1.93	1.43
	SD	1.04	0.95	1.03	0.96	1.30	0.99	0.92	1.02

F-value		0.607	**2.935**	0.463	1.260	0.437	1.593	1.393	0.342
p-value		n.s.	**0.033**	n.s.	n.s.	n.s.	n.s.	n.s.	n.s.

Christian denomination	Mean	0.80	0.80	1.33	1.66	2.07	2.51	1.85	1.46
	SD	1.02	0.97	1.22	1.30	1.30	1.21	1.22	1.17
None	Mean	0.69	0.73	1.16	1.47	1.70	1.97	1.97	1.46
	SD	0.98	0.96	1.23	1.32	1.43	1.44	1.43	1.36

F-value		0.724	**14.346**	1.522	1.850	1.003	**6.364**	0.406	0.001
p-value		n.s.	**0.000**	n.s.	n.s.	n.s.	**0.012**	n.s.	n.s.

Breast cancer	Mean	0.72	0.75	1.25	1.55	1.99	2.37	1.89	1.46
	SD	0.97	0.96	1.23	1.33	1.34	1.30	1.30	1.25
Colorectal cancer	Mean	1.08	1.00	1.23	1.71	1.69	1.71	1.58	1.62
	SD	1.26	0.91	1.01	0.99	1.03	1.07	0.90	0.96
Ovarian cancer	Mean	0.38	0.54	1.08	2.00	1.77	2.31	1.23	1.15
	SD	0.51	0.78	1.32	1.35	1.69	1.65	1.09	1.14
Other localizations	Mean	1.13	0.93	1.47	1.82	1.87	2.34	2.07	1.38
	SD	1.16	1.04	1.20	1.21	1.47	1.38	1.34	1.19

F-value		1.650	1.093	0.514	1.038	**3.365**	0.389	0.935	0.397
p-value		n.s.	n.s.	n.s.	n.s.	**0.019**	n.s.	n.s.	n.s.

There were no significant differences with respect to the family status, educational level, duration of disease and Body Mass Index

Patients with breast cancer had significantly higher ratings for the perception 'value' than patients with colorectal or ovarian cancer (Table 3). Although mainly rejected, the ratings of the interpretations 'weakness' (F = 4.731; p = 0.031) and 'punishment' (F = 4.133; p = 0.044) were slightly higher in patients with a relapse of their disease, while patients with dissemination had lower ratings for 'call for help' (F = 3.920; p = 0.050) and 'value' (F = 2.944; p = 0.089).

To analyze the impact of covariates (and their interactions), i.e., age group, family status, educational level, religious denomination and tumor localization, we performed univariate analyses (GLM univariate, between subject effects). There were just some remarkable trends for complex pattern of variables which involved in most cases religious affiliation, educational level and tumor localization (Table 4).

**Table 4:** Influence of covariates (and interdependencies) on disease perception (GLM univariate, between subject effects)

**Dependent variables**	Variables *	**F-value**	Significance **
Weakness/failure	educational level * localization	2.230	0.033

Punishment	educational level * localization	2.256	0.031

Relieving break	Denomination * educational level * localization	5.175	0.024

Call for help/strategy	Denomination * age group * family status	3.294	0.039

Value	Denomination * educational level * localization	4.691	0.031

Challenge	Denomination localization	5.700 2.765	0.018 0.043

Interruption/loss	Denomination * educational level Family status * educational level * localization	3.177 3.131	0.014 0.045

Enemy/threat	Denomination * educational level	2.740	0.029

* Only significant variables were presented.

** Levene's test for equality of variances was significant in all cases and thus the level of significance should be p < 0.01.

### Correlations between Interpretation of Illness and health-related variables

To make statements about the conceptual relationships between disease interpretations and health related variables, we performed correlation analyses (Table 5). Both mental health-related quality of life and life satisfaction were negatively associated with the negative interpretations 'loss' and 'enemy', but also with 'call for help', while physical health did not correlate with any of the disease interpretations.

**Table 5:** Correlations between Meaning of Illness, adaptive coping and health related variables

	**Weak-ness/failure**	**Punishment**	**Relieving break**	**Call for help/strategy**	**Value**	**Challenge**	**Interrupt/loss**	**Enemy/threat**
	*guilt-associated negative*		*strategy-associated*		*positive*		*fatalistic negative*	

**Health-related quality of life**								

SF-12's physical health	-.013	-.018	-.032	-.032	.068	-.057	-.125	-.042
SF-12's mental health	-.132	-.135*	-.105	-.196*	.028	.002	-.254*	-.216*

**Anxiety**	.156*	.203*	.168*	.232*	-.047	.049	.198*	.287*
**Depression**	.102	.108	.140*	.184*	-.102	-.014	.172*	.250*
**Fatigue**	.059	.035	.086	.125	-.047	.010	.105	.142*

**Life Satisfaction**	-.112	-.166*	-.120	-.208*	.055	.057	-.213*	-.223*

**Adaptive coping strategies**								

Conscious and Healthy Living	-.100	-.160*	-.068	-.009	.137*	.189*	-.022	-.102*
Positive Attitudes	-.028	-.080	.016	.040	.155*	.208*	.039	-.018
Reappraisal: Illness as Chance	.117	.009	.290*	.310*	.477*	.539*	-.030	.010
Trust in Medical Help	-.189*	-.147*	-.030	.051	.062	.159*	.010	.068
Search for Information and Alternative Help	-.006	-.040	.111	.138*	.135*	.258*	.130*	.138*
Trust in God's Help	.077	.044	.123	.125	.227*	.298*	-.014	.044

Escape from Illness	.235*	.253*	.197*	.103	-.038	.043	.298*	.378*

* p < 0.01 (Pearson, 2-tailed).

Anxiety was moderately associated with all negative ratings, particularly with 'enemy/threat'. Depression correlated significantly with strategy-associated interpretations and with fatalistic negative interpretations (particularly with 'enemy/threat'), while fatigue correlated weakly just with 'enemy'.

Among the adaptive coping strategies, the positive interpretations correlated well with Reappraisal: Illness as Chance, particularly 'challenge' (r = 0.54) and 'value' (r = 0.48). 'Challenge' correlated (moderately) also with Trust in God's Help, with Search for Information and Alternative Help, and with Positive Attitudes. In contrast, Escape from Illness (avoidance/escape strategy) correlated moderately with the negative interpretations, particularly with 'enemy' (r = 0.38).

If one analyses patients with positive ratings for 'challenge' and 'interruption' as compared to patients lacking these interpretations, it becomes clear that patients with a 'challenge' perception had a significantly higher utilization of adaptive coping styles (Table 6), while their health-related quality of life, anxiety, depression, fatigue, Escape from illness and life satisfaction did not significantly differ from patients without this perception (Table 7). In contrast, patients with an 'interrupt/loss' attitude had significantly lower health-associated scores (Table 7), while their utilization of adaptive coping strategies did not differ from the patients without this 'loss' perception (Table 6).

**Table 6:** Adaptive coping strategies in patients with disease interpretations Challenge and Interruption

Independent variables		**Conscious and Healthy Living**	**Positive Attitudes**	**Reappraisal: Illness as Chance**	**Trust in Medical Help**	**Search for Information and Alternative Help**	**Trust in God's Help**
Challenge: No	Mean	79.17	74.41	50.18	75.30	67.40	47.33
(scores ≤ 2)	SD	13.31	16.24	23.57	18.20	18.99	34.30
**Challenge: Yes**	Mean	83.25	80.32	72.67	83.34	76.56	64.66
(scores > 2)	SD	10.50	14.62	20.29	24.76	17.13	33.27

total	Mean	81.33	77.53	62.10	79.55	72.24	56.49
(scores 0–4)	SD	12.07	15.66	24.58	22.25	18.58	34.81

F-value		**11.321**	**14.148**	**101.170**	**12.969**	**24.836**	**25.229**
p-value		**0.001**	**0.000**	**0.000**	**0.000**	**0.000**	**0.000**

Interruption: No	Mean	81.59	76.79	63.37	78.88	70.75	57.75
(scores ≤ 2)	SD	12.12	15.02	24.93	24.33	19.25	35.18
**Interruption: Yes**	Mean	80.52	78.49	59.07	80.28	74.04	53.81
(scores > 2)	SD	12.12	16.86	24.23	18.07	17.44	34.23

Total	Mean	81.21	77.39	61.85	79.37	71.91	56.36
(scores 0–4)	SD	12.11	15.69	24.74	22.31	18.68	34.85

F-value		0.671	1.035	2.642	0342	2.731	1.114
p-value		n.s.	n.s.	n.s.	n.s.	0.099	n.s.

**Table 7:** Health-related variables in patients with disease perception Challenge and Interruption

Independent variables		**Physical health**	**Mental health**	**Anxiety**	**Depression**	**Escape from Illness**	**Fatigue**	**Life satisfaction**
Challenge: No	Mean	43.60	46.72	6.62	4.55	37.61	24.85	75.01
(scores ≤ 2)	SD	9.41	10.25	3.73	3.54	23.22	9.89	17.14
**Challenge: Yes**	Mean	41.94	47.30	6.84	4.37	37.05	25.77	76.28
(scores > 2)	SD	9.96	10.06	3.62	3.34	24.47	9.40	14.65

total	Mean	42.71	47.03	6.74	4.46	37.32	25.34	75.68
(scores 0–4)	SD	9.73	10.14	3.67	3.43	23.86	9.64	15.87

F-value		2.725	0.299	.354	.257	0.054	0.868	0.609
p-value		n.s.	n.s.	n.s.	n.s.	n.s.	n.s.	n.s.

Interruption: No	Mean	43.79	48.60	6.35	4.06	33.80	24.45	77.47
(scores ≤ 2)	SD	9.45	9.81	3.54	3.39	23.08	9.26	16.17
**Interruption: Yes**	Mean	40.66	44.15	7.47	5.19	43.80	27.12	72.25
(scores > 2)	SD	9.90	10.12	3.78	3.40	23.95	9.92	14.75

total	Mean	42.66	47.00	6.75	4.46	37.33	25.39	75.63
(scores 0–4)	SD	9.72	10.14	3.66	3.43	23.84	9.57	15.86

F-value		**9.071**	**17.161**	**8.276**	**9.759**	**15.954**	**6.888**	**9.648**
p-value		**0.003**	**0.000**	**0.004**	**0.002**	**0.000**	**0.009**	**0.002**

## Discussion

Our results enrolling cancer survivors (all female, only one male) confirmed the findings of others that 'challenge' and 'value' were rated highest by cancer patients [7-9]. However, in our study the patients were advised to make a statement to each of the eight categories, and thus, also the negative interpretation 'interruption of life/loss' was rated high (35%) – which was rated only by 4% of the Canadian [7] and by 16% of the Swedish population [9]. A Christian denomination was associated with the 'challenge' perception, while particularly 'punishment' was rated lower in younger patients and individuals without a religious affiliation. In previous studies we have observed a positive association between spirituality/religiosity and a positive interpretation of disease in terms of reflection and change of life [19,21,22], which could explain the linkage between religious denomination and this positive disease interpretation.

Although the subjective interpretation of illness is influenced by intrapersonal, disease-related and environmental factors, the results of Wallberg et al. [9] and ours indicate that neither the educational level nor time from diagnosis had an impact on the disease interpretations, while age and religious denomination had an influence. In our study, particularly elderly had higher 'challenge' scores than younger patients, while in the Swedish cancer patients 'challenge' was rated less frequently by elderly. These differences can not really be explained.

It is striking that several categories defined by Lipowski are not necessarily identically with the interpretations of the patients interviewed by Wallberg et al. [9], i.e., 'strategy' was interpreted "close to challenge" or "means changes" and adaptation to new situations [9]; 'relief' was interpreted in terms of clear diagnostic and therapeutic decisions. Thus, the conceptual value of these items remains unclear.

We performed principal component analysis to clarify the factorial structure of the categories. Although the factorial structure of IIQ items shares similarities with the category grouping of Degner et al. [7] and Wallberg et al. [9] (i.e., Challenge; Enemy/Punishment/Weakness/Irreparable Loss; Value/Relief/Strategy), there were some conceptually relevant differences. Exploratory factor analysis indicated 3 main factors. Among them, two are sound from a theoretical point of view (i.e., the 'fatalistic' negative interpretations 'interruption of life/loss' and 'enemy/threat' on the one hand, and the positive interpretations 'challenge' and 'value' on the other hand). However, the main factor comprised two conceptually different topics, i.e., the guilt-associated negative interpretations 'weakness/failure' and 'punishment' on the one hand, and the strategy-associated items 'relieving break' and 'call for help' on the one hand. These strategy-associated interpretations can be both negative and positive, depending on the individual interpretation.

Although the instrument as a differential questionnaire is in need of further refinement, the 8-item schema nevertheless seems to be of value. Also Degner et al. stated the usefulness of a brief measure of meaning that can be incorporated into surveys [7]. Of relevance is the fact that the 'fatalistic' negative interpretations are inversely correlated with mental health-related quality of life and life satisfaction, and positively with an escape-avoidance strategy, depression and anxiety. The guilt-associated negative interpretations correlated best with Escape from illness, moderately with anxiety, but not with depression. Thus, among the negative meanings, one has to state differential intercorrelations with mental health-associated variables. In contrast, the positive disease interpretations correlated only with adaptive coping strategies, particularly with Reappraisal and Trust in God's Help. An association between reappraisal as a coping strategy and reliance on an external (divine) locus of disease/health control was confirmed previously, particularly for patients with cancer [19]. It reflects the aforementioned spiritual connotation of the unique view that illness can be regarded as a 'chance' to reflect and change of life. However, healthy individuals do not share the attitude that illness could be regarded as a 'chance' or 'challenge'; it is unique point of view found more often in cancer patients than in other patients with chronic diseases [19]. One may speculate that it reflects the struggle with the subliminal fear that the disease may return again, even after a putative 'effective' treatment; in the light of this constant stressful insecurity, the patients have to find strategies to adapt. One possibility to behave could be to rely on a more powerful (divine) external help (in terms of religious coping), which was found particularly in elderly and patients with cancer [4,19,21,22], and to focus on active adaptive coping strategies (in terms of internal locus of disease control) – which indeed were highly utilized in patients with chronic diseases [19].

Despite a fatal diagnosis and unclear prognosis, several cancer patients nevertheless regarded their illness as a challenge, others as an interruption of life. Both interpretations are exclusive and do not correlate (r = 0.09). According to Lipowski's original thesis, the experience of illness may enhance intensity and depth of life [6]. Indeed, particularly the positive interpretations were associated with active and adaptive coping strategies to deal with the chronic disease. This is in line with Antonovsky's 'Sense of Coherence' concept [23], i.e., patients find access to resources required to meet the demands and are willing to search them out ('manageability'). This means, health is a changing continuum and patients have to adapt their strategies to changing situations. This implies that the strategies and also the interpretations of disease may change during the course of illness. In our analysis, we did not find significant differences with respect to the duration of disease (albeit this may be different in the acute phase of illness). In the Canadian [6] and also in the Swedish population [9], the stage of disease was a powerful factor. In the German cancer patients, the guilt-associated negative interpretations 'weakness' and 'punishment' were less frequently rated; particularly in patients with a relapse, the relevance of these interpretations showed some minor variances. In contrast, patients with dissemination had in trend slightly lower ratings for the interpretations 'call for help' and 'value'. As a matter of fact, patients who know that they are in a progressive state of disease will interpret their illness more negatively than positively.

Although one may argue that it is a limitation of the study that we have focused on cancer survivors rather than patients during early phases of the disease, we believe that this study nevertheless may fill a gap of knowledge how these 'late phase' patients do behave and interpret their illness.

## Conclusion

Despite factorial limitations, the 8-item schema of the IIQ may provide a useful screening approach to identify patients at risk for reduced psychosocial functioning. Both the inverse correlations between the negative interpretations and health-related variables, and the associations between positive meanings and adaptive coping strategies may underline the need for further research and appropriate supportive care interventions. Changing negative illness interpretations and depressive or avoidance coping by means of an intervention and encouraging social support by means of patient support groups may at least improve quality of life. Further studies should focus on the clinical relevance of psycho-oncological interventions with respect to disease interpretation, decision making and quality of life.

## Competing interests

The authors do not have any competing interests, and did not receive a grant for this analysis. The results are part of JF's doctoral thesis.

## Authors' contributions

AB designed the questionnaire according to Lipwski's concept "Meaning of Illness", performed statistical analysis and drafted the manuscript. JF recruited the patients and participated to draft the manuscript. All authors read and approved the final manuscript.

## Pre-publication history

The pre-publication history for this paper can be accessed here:



## References

[B1] Folkman S, Lazarus RS (1980). An analysis of coping in a middle-aged community sample. J Health Soc Behav.

[B2] Carver CS, Scheier MF, Weintraub JK (1989). Assessing coping strategies: a theoretically based approach. J Pers Soc Psychol.

[B3] Holahan CJ, Moos RH (1987). Risk, resistance, and psychological distress: a longitudinal analysis with adults and children. J Abnorm Psychol.

[B4] Derks W, Leeuw JR, Hordijk GJ, Winnubst JA (2005). Differences in coping style and locus of control between older and younger patients with head and neck cancer. Clin Otolaryngol.

[B5] Lipowski ZJ (1983). Psychosocial reactions to physical illness. Can Med Assoc J.

[B6] Lipowski ZJ (1970). Physical illness, the individual and the coping processes. Psychiatry in Medicine.

[B7] Degner LF, Hack T, O'Neil J, Kristjanson LJ (2003). A new approach to eliciting meaning in the context of breast cancer. Cancer Nurs.

[B8] Luker KA, Beaver K, Leinster SJ, Owens RG (1996). Meaning of illness for women with breast cancer. J Adv Nurs.

[B9] Wallberg B, Michelson H, Nystedt M, Bolund C, Degner L, Wilking N (2003). The meaning of breast cancer. Acta Oncol.

[B10] Büssing A, Keller N, Michalsen A, Moebus S, Ostermann T, Matthiessen PF (2006). Spirituality and Adaptive Coping Styles in German Patients with Chronic Diseases in a CAM Health Care Setting. Journal of Complementary and Integrative Medicine.

[B11] Caress AL, Luker KA, Owens RG (2001). A descriptive study of meaning of illness in chronic renal disease. J Adv Nurs.

[B12] Resnick B, Nahm ES (2001). Reliability and validity testing of the revised 12-item Short-Form Health Survey in older adults. J Nurs Meas.

[B13] Ware J, Kosinski M, Keller SD (1996). A 12-Item Short-Form Health Survey: construction of scales and preliminary tests of reliability and validity. Med Care.

[B14] Bssing A, Fischer J, Haller A, Heusser P, Ostermann  T, Matthiessen  PF (2009). Validation of the Brief Multidimensional Life Satisfaction Scale in patients with chronic diseases. European Journal of Medical Research.

[B15] Bower JE (2008). Behavioral symptoms in patients with breast cancer and survivors. J Clin Oncol.

[B16] Herrmann C (1997). International experiences with the Hospital Anxiety and Depression Scale – a review of validation data and clinical results. J Psychosom Res.

[B17] Zullig KJ, Huebner ES, Gilman R, Patton JM, Murray KA (2005). Validation of the brief multidimensional students' life satisfaction scale among college students. Am J Health Behav.

[B18] Kröz M, Zerm R, Reif M, Laue HBv, Schad F, Büssing A (2008). Validation of the German version of the Cancer Fatigue Scale (CFS-D). Eur J Cancer Care (Engl).

[B19] Büssing A, Ostermann T, Matthiessen PF (2007). Adaptive coping and spirituality as a resource in cancer patients. Breast Care.

[B20] Büssing A, Matthiessen PF, Mundle G (2008). Emotional and rational disease acceptance in patients with depression and alcohol addiction. Health Qual Life Outcomes.

[B21] Büssing A, Ostermann T, Matthiessen PF (2005). Search for meaningful support and the meaning of illness in German cancer patients. Anticancer Res.

[B22] Büssing A, Ostermann T, Koenig HG (2007). Relevance of religion and spirituality in German patients with chronic diseases. Int J Psychiatry Med.

[B23] Antonovsky A (1987). Unraveling the mystery of health How people manage stress and stay well.

